# Association of Geography and Ambient Air Pollution with Urine Metal Concentrations in Six US Cities: The Multi-Ethnic Study of Atherosclerosis

**DOI:** 10.3390/ijerph13030324

**Published:** 2016-03-15

**Authors:** Yuanjie Pang, Miranda R. Jones, Maria Tellez-Plaza, Eliseo Guallar, Dhananjay Vaidya, Wendy S. Post, Joel D. Kaufman, Joseph A. Delaney, Ana Navas-Acien

**Affiliations:** 1 Departments of Epidemiology, Johns Hopkins University Bloomberg School of Public Health, Baltimore, MD 21205, USA; mjone132@jhu.edu (M.R.J.); eguallar@jhu.edu (E.G.); wpost@jhmi.edu (W.S.P.); anavasa1@jhu.edu (A.N.-A.); 2 Departments of Environmental Health Sciences, Johns Hopkins University Bloomberg School of Public Health, Baltimore, MD 21205, USA; mtellezp@gmail.com; 3 Fundacion de Investigacion Hospital Clinico de Valencia INCLIVA, Valencia 46010, Spain; 4 Department of Medicine, Johns Hopkins School of Medicine, Baltimore, MD 21205, USA; dvaidya@jhmi.edu; 5 Welch Center for Prevention, Epidemiology and Clinical Research, Johns Hopkins University, Baltimore, MD 21205, USA; 6 Department of Environmental and Occupational Health Sciences, School of Public Health, University of Washington, Seattle, WA 98195, USA; joelk@u.washington.edu; 7 Department of Epidemiology, School of Public Health, University of Washington, Seattle, WA 98195, USA; jacd@u.washington.edu

**Keywords:** metals, geography, air pollution, exposure modeling

## Abstract

We investigated the associations of urinary concentrations of antimony, cadmium, tungsten and uranium with geographic locations and with ambient air pollution in 304 adults in the Multi-Ethnic Study of Atherosclerosis from six US cities. After adjustment for sociodemographics, body mass index, and smoking status, urinary cadmium was the highest in Winston-Salem among all study sites (the geometric mean [GM] in Winston-Salem was 0.84 µg/L [95% confidence interval (CI) 0.57–1.22]). The adjusted GMs of urinary tungsten and uranium were highest in Los Angeles (0.11 µg/L [95% CI 0.08–0.16] and 0.019 µg/L [95% CI 0.016–0.023], respectively). The adjusted GM ratio comparing fine particulate matter (PM_2.5_) tertiles 2 and 3 with the lowest tertile were 1.64 (95% CI 1.05–2.56) and 3.55 (95% CI 2.24–5.63) for tungsten, and 1.18 (95% CI 0.94–1.48) and 1.70 (95% CI 1.34–2.14) for uranium. The results for tungsten remained similar after adjustment for study site. Urinary cadmium, tungsten and uranium concentrations differed by geographic locations in MESA (Multi-Ethnic Study of Atherosclerosis) communities. PM_2.5_ levels could contribute to geographic differences in tungsten exposure. These findings highlight the need to implement preventive strategies to decrease toxic metal exposure and to evaluate the health effects of chronic exposure to those metals.

## 1. Introduction

Exposure to metals is widespread in the environment. Experimental and epidemiologic evidence support that low-to-moderate chronic exposure to certain toxic metals plays a role in the development of cardiovascular disease, kidney disease, neurocognitive outcomes and also some cancers [1,2,3,4,5]. Cadmium has been associated with cardiovascular disease, chronic kidney disease, and some cancers [2,3,4,5]. Antimony and tungsten have been linked to cardiovascular disease, peripheral arterial disease, and diabetes [2,6,7]. Uranium compounds are associated with chronic kidney disease and cancer in occupationally exposed populations [8,9], whereas a recent study from the National Health and Nutrition Examination Survey (NHANES) reported that higher levels of urinary uranium were associated with diabetes [7].

Antimony, cadmium, tungsten and uranium are naturally present in the earth’s crust, and are released to the environment from various anthropogenic sources [10,11,12,13]. Smoking roughly doubles cadmium body burden in comparison to never smoking [12,14], while secondhand smoke is also a relevant source [14]. In 2000, the state-specific prevalence of current cigarette smoking among adults in the US was markedly high in North Carolina (26.1%), while they were relatively low in Illinois, Maryland, Minnesota, New York (range 19.8%–22.3%), and California (17.2%) [15]. Groundwater can be a source of tungsten and uranium exposure especially in certain geographical areas that contain higher levels of tungsten and uranium in the rocks and soil, including the Western US [11,13]. California is the state with the third highest uranium concentrations in drinking water, with uranium levels of 2.7 pCi/L [16]. However, tungsten levels in drinking water are generally unknown [11]. In ambient air, tungsten exists in the particulate phase. Tungsten enters into the air during wind erosion, tungsten ore processing, alloy fabrication, tungsten carbide production and use, and municipal waste combustion [17,18]. Uranium can be released into the air through wind erosion, volcanic eruptions, and industries involved in mining, milling, and processing of uranium [13].

Previous studies using data from the NHANES on urinary and blood metals have focused on sociodemographics, dietary differences, and on changes over time [14,19,20]. Place of residency, however, might be an important source of variation for metal exposures as natural and anthropogenic sources of metals are different across geographic sites. Airborne metals can result in inhalation and possible ingestion of metals, contributing to increased metal body burden [21,22]. Previous studies have shown that particulate matter concentrations were associated with urinary cadmium in populations living in contaminated areas [23] and with uranium in occupational populations [24]. Evidence for the associations of ambient air pollution with urinary metals in the general population is limited.

The objectives of this study were to evaluate the geographical differences in urinary concentrations of antimony, cadmium, tungsten and uranium, and to evaluate the association of air pollution levels (fine particulate matter (PM_2.5_) and nitrogen oxides (NOx; sum of nitric oxide, nitrogen dioxide, nitrous acid, and nitric acid)) with urinary antimony, cadmium, tungsten and uranium. The reasons for selection of these four metals include their possible health impact reported from previous studies, availability of urine metal measures in our study population, and the acceptance of urine as biomarkers of exposure to these metals. We focused this analysis on PM_2.5_ and NO_X_ because PM_2.5_ reflects overall exposure to fine particles, whereas NO_X_ is a good marker of traffic-related air pollution, which might capture a complex mixture of combustion byproducts including metals [21].

We hypothesized that participants from Los Angeles would have higher urinary uranium due to higher uranium concentrations in drinking water in the Western US including California [13], and that participants from Winston-Salem would have higher urinary cadmium because the smoking prevalence is higher and the North Carolina law does not mandate 100% smoke free non-hospitality workplaces, restaurants, and bars [25]. For the association between metals and ambient air pollutants (PM_2.5_ and NOx), we had no *a priori* hypotheses as little is known regarding the contribution of air pollution to metal exposure. The use of a multi-ethnic cohort that recruited participants from six different cities across the US allowed us to evaluate previously unexplored differences in metal exposures in geographically diverse populations. This study may also support the need to conduct a larger study to evaluate a range of health effects associated with these metals in communities of the Multi-Ethnic Study of Atherosclerosis.

## 2. Materials and Methods

### 2.1. Study Population

The Multi-Ethnic Study of Atherosclerosis (MESA) is a population-based cohort study evaluating risk factors for atherosclerosis progression and cardiovascular disease development in participants aged 45 to 84 years old who were free of cardiovascular disease at baseline (2000–2002) in 6 urban communities in the United States (Baltimore, MD; Chicago, IL; Los Angeles, CA; New York, NY; St. Paul, MN; and Winston-Salem, NC) [26]. We recently measured baseline urinary metal concentrations in an overall sample of 310 participants from the 6 study sites based on funding available (90 White, 75 Black, 75 Hispanic, and 70 Chinese American participants). These 310 participants were selected using random stratification by site and race group with a predetermined distribution of participants per race and site to ensure sufficient numbers for stratified analyses. For this study, we excluded 6 participants missing data on ambient air pollution exposures, leaving a total of 304 participants for this analysis (Table 1). The MESA study was approved by the institutional review boards of each study site and written informed consent was obtained from all participants.

Antimony, cadmium, tungsten and uranium were measured in spot urine specimens collected at MESA Exam 1 (2000) using inductively coupled plasma mass spectrometry (ICPMS) at the Trace Element Laboratory of University of Graz, Austria following an established protocol [27]. The limits of detection (LOD) were 0.006 µg/L for antimony, 0.015 µg/L for cadmium, 0.005 µg/L for tungsten, and 0.008 µg/L for uranium. In total, 11.2%, 2.3%, 24.3%, and 34.5% of sample levels were below the LOD for antimony, cadmium, tungsten, and uranium, respectively. For those samples below the LOD, we replaced their values by the LOD divided by the square root of two. To account for urine dilution, urinary metals were all adjusted for urinary specific gravity according to urinary metal concentration * (mean urinary specific gravity − 1)/(urinary specific gravity − 1) [28]. Estimated glomerular filtration rate (eGFR) was calculated from recalibrated creatinine, age and sex using the Chronic Kidney Disease Epidemiology Collaboration (CKD-EPI) formula [29].

The Multi-Ethnic Study of Atherosclerosis and Air Pollution (MESA Air) generated predictions of individual-level long-term ambient concentrations of PM_2.5_ and NOx for MESA participants as described elsewhere [30]. Baseline air pollution concentrations at each participant’s home address were predicted using area-specific hierarchical spatio-temporal models [30,31,32]. These models utilize spatially-varying long-term average concentrations, seasonal and long-term trends, and spatially-correlated, but temporally-independent residuals. The MESA Air exposure models are built using monitoring data from the US Environmental Protection Agency (EPA) Air Quality System, supplemented with monitors deployed by MESA Air at fixed sites throughout the study area, monitors at participants’ homes, and monitors placed at specific locations to capture roadway concentration gradients (especially in the NOx models). The study-specific data were collected from 27 fixed site monitors situated in MESA communities which collected over 100 consecutive 2-week integrated air samples over the course of the study; monitors placed at a subset of nearly 700 participant homes; and, for NOx monitoring, during simultaneous deployment (“snapshot” campaigns) of over 100 samples in each MESA region during each of three seasons [33]. Each model also employed geographic variables including roadway density, land use, and outputs from dispersion models. To characterize ambient air pollution exposure in this study, we used likelihood-based annual average concentrations of PM_2.5_ and NOx for the year 2000 that were estimated for each participant based on the location(s) lived during that year.

Demographics and dietary intake were assessed by questionnaires at MESA Exam 1 (2000) [26]. Participant race/ethnicity was categorized as non-Hispanic White (“White”), non-Hispanic Black (“Black”), Hispanic and Chinese American. Participant education was measured as the highest level completed and categorized as less than or equal to high school and more than high school. Annual family income was collected in thirteen categories and categorized as <$25,000, $25,000–50,000, $50,000–75,000, and ≥$75,000. Smoking status was defined as current, former, and never. Pack-years were calculated by multiplying the reported average number of packs of cigarettes smoked per day by the number of years of smoking. Body mass index (BMI) was calculated by dividing measured weight in kilograms by measured height in meters squared. Information on the usual dietary intake during the past year was assessed using a 120-item food frequency questionnaire. We used the 47 MN food groups developed by the working group at the University of Minnesota [34]. Approximately 4.7% of the data elements in the baseline MESA food frequency diet data were missing, and this missingness was accounted for with imputation techniques [34].

### 2.2. Statistical Analysis

Descriptive analyses were conducted by study site and by participant characteristics. Urinary antimony, cadmium, tungsten and uranium were all right skewed and log transformed for the analysis.

We used multivariable linear regression model with log-transformed urinary metal as the dependent variable. To assess the distribution of metal exposure across study site, we performed pairwise comparisons across the six study sites and calculated adjusted geometric means (GM) at representative values of covariates. For each participant, education was set to “more than high school”, annual family income to “$50,000–75,000”, race/ethnicity to White, and smoking to former smoker. Other covariates were set to their mean values. To calculate GMs, the predicted log-transformed values of urinary metals were averaged over the participants from each study site and back transformed. We initially adjusted for age, sex, race/ethnicity, BMI, education, and annual family income (Model 1). In Model 2, we further adjusted for smoking status and pack-years. In Models 3 and 4, we further adjusted for log-transformed PM_2.5_ and NOx, respectively. We also computed GM ratios of these metals by comparing each tertile to the bottom tertile of PM_2.5_ and NOx, respectively. We used the same Models 1 and 2 as in the site analysis. In Model 3, we further adjusted for the other pollutant (log-PM_2.5_ for NOx and log-NOx for PM_2.5_) (two-pollutant model). Model 4 was Model 2 with additional adjustment for study site (categorical). P-value for linear trend was obtained by including in the regression model a continuous variable with the medians corresponding to each tertile of the PM_2.5_/NOx distribution.

We ran several sensitivity analyses. First, we repeated the analysis of urinary cadmium and study site in all four models with further adjustment for secondhand smoke using self-reported hours per week of current secondhand smoke exposure in non-current smokers (see Appendix: Table A1). This is because that secondhand smoke is also a potential source of exposure to cadmium besides active smoking (this model was run as a sensitivity analysis because there were 39 participants missing secondhand smoke exposure information) [12]. Second, we conducted the analysis of urinary cadmium and study site with further adjustment for food with high cadmium levels (fruits, vegetables, grains, nuts, red meat and shellfish) [35,36], with similar results (see Appendix: Table A1). The aim was to control for the impact of the difference in dietary cadmium intake on the geographic associations. Third, we additionally included baseline eGFR in every model to account for the between-site difference in kidney function and its possible impact on the associations evaluated, with similar results (see Appendix: Table A2, Table A3 and Table A4). Fourth, urinary tungsten and uranium levels below the LOD were imputed using sequential regression implemented in Stata 13.0 using the Multiple Imputation (MI) program [37], with similar results (see Appendix: Table A5, Table A6 and Table A7). Both urinary tungsten and uranium were imputed as a function of age, sex, race/ethnicity, education, annual family income, and study site and the number of imputations was 10. Last, we ran stratified analyses by sex to evaluate possible sex differences in the geographic associations and the associations with air pollution, with similar results (see Appendix: Table A8, Table A9, Table A10, Table A11, Table A12 and Table A13). All statistical analyses were performed using Stata version 13.0 (StataCorp LP, College Station, TX, USA). All statistical tests were 2-sided and confidence intervals were set at 95%.

## 3. Results

### 3.1. Metal Levels in Urine

**Figure 1:**
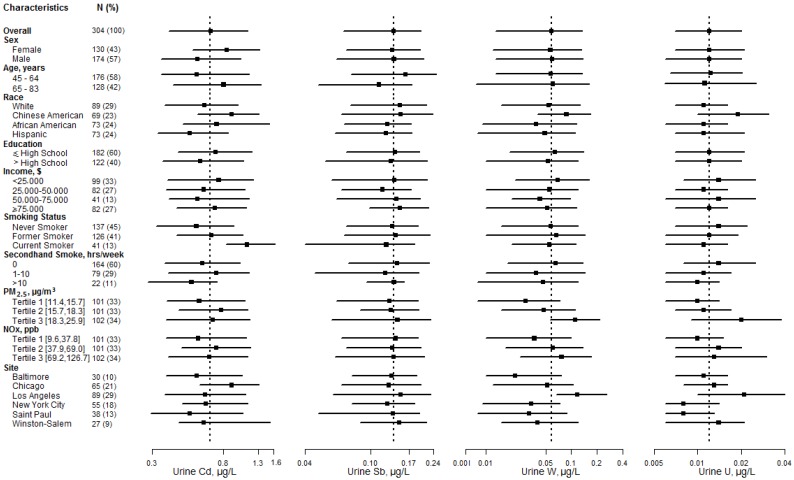
Metal levels in urine (µg/L) by participant characteristics. Horizontal lines, interquartile ranges; squares, medians; dotted vertical line, the geometric mean for the overall study sample.

The mean age ranged from 58.5 years in Saint Paul to 63.4 years in New York City, and the percentage of men ranged from 40.0% in Winston-Salem to 67.7% in Chicago (Table 1). Women had higher levels of cadmium than men, and levels of antimony tended to decrease with age (Figure 1). Compared with Whites, Chinese Americans had higher levels of tungsten and uranium. Compared with participants in Winston-Salem, participants in Los Angeles had higher levels of tungsten and uranium. Both levels of tungsten and uranium increased with PM_2.5_ tertiles. Levels of tungsten and uranium also increased with NOx tertiles, but to a lesser extent than with PM_2.5_.

### 3.2. Urinary Metals and Study Sites

Across the six study sites in MESA, the adjusted GM of urinary cadmium was highest in Winston-Salem (0.84 µg/L, 95% CI: 0.57–1.22, p value with Bonferroni correction comparing Winston-Salem to Baltimore 0.73; Model 2 in Table 2). Further adjustment for PM_2.5_ or NOx did not change the GMs much (Models 3 and 4 in Table 2). In a sensitivity analysis with further adjustment for secondhand smoke, the adjusted GM of urinary cadmium in Winston-Salem decreased by 26.2% (GM 0.62 µg/L, 95% CI: 0.51–0.76; Model 1 in Appendix: Table A1), suggesting an attenuation in urinary cadmium after adjustment for self-reported secondhand smoke exposure. The adjusted GMs of urinary antimony were similar across study sites in MESA in all models.

The adjusted GM of urinary tungsten was highest in Los Angeles among all study sites (0.11 µg/L, 95% CI 0.08, 0.16, *p* value with Bonferroni correction comparing Los Angeles to Saint Paul < 0.001; Model 2 in Table 2). Further adjustment for PM_2.5_ attenuated the GM in Los Angeles by 36.4% (0.070 µg/L, 95% CI: 0.035–0.14; Model 3 in Table 2), while adjustment for NOx attenuated the GM by 12.7% (0.096 µg/L, 95% CI: 0.063–0.15; Model 4 in Table 2). The adjusted GM of urinary uranium was highest in Los Angeles among all six sites (0.019 µg/L, 95% CI: 0.016–0.023, *p* value with Bonferroni correction comparing Los Angeles to Saint Paul < 0.001; Model 2 in Table 2). Further adjustment for either PM_2.5_ or NOx did not change the GMs much (Models 3 and 4 in Table 2).

### 3.3. Urinary Metals and Ambient Air Pollution

Urinary tungsten was positively associated with PM_2.5_ levels (Table 3). After adjustment for age, sex, race/ethnicity, BMI, education, annual family income, smoking status, and pack-years, GM ratios of urinary tungsten comparing PM_2.5_ tertiles 2 and 3 with the lowest tertile were 1.64 (95% CI: 1.05–2.56) and 3.55 (95% CI: 2.24–5.63) (Model 2 in Table 3). Significant but weaker associations were observed after further adjustment for study site (Model 4 in Table 3). Urinary uranium was only weakly associated with PM_2.5_ levels (Table 3). The adjusted GM ratios of urinary uranium comparing PM_2.5_ tertiles 2 and 3 with the lowest tertile were 1.18 (95% CI: 0.94–1.48) and 1.70 (95% CI: 1.34–2.14) (Model 2 in Table 3). When we further adjusted for study site, the magnitude of association decreased and included the null value (Model 4 in Table 3).

Association of NOx with urinary tungsten and uranium only existed in minimally adjusted models (Model 1 and Model 2 in Appendix: Table A14). When further adjusted for PM_2.5_, the observed associations disappeared. For urinary antimony and cadmium, no association was apparent with either PM_2.5_ or NOx (Table 3 and Appendix: Table A14).

## 4. Discussion

Urinary cadmium, tungsten and uranium concentrations differed by geographic locations in MESA communities. Participants from Winston-Salem had higher urinary cadmium levels than participants from any other site. Higher cadmium concentrations in Winston-Salem could reflect the higher prevalence of active smoking and possibly the higher secondhand smoke exposure. Participants from Los Angeles had higher urinary tungsten and uranium levels than participants from any other site. Higher tungsten and uranium levels in Los Angeles could reflect the higher exposure from groundwater. PM_2.5_ levels were associated with higher urinary tungsten and uranium levels. The association between PM_2.5_ and urinary tungsten levels decreased but persisted after further adjustment for study site, whereas the association between PM_2.5_ and urinary uranium was markedly attenuated and became nonsignificant. PM_2.5_ levels could contribute to the geographic difference in tungsten exposure. Although sex differences in urinary metal concentration levels may exist due to physiological status and sociobehavioral factors, overall we observed consistent associations by geography and PM_2.5_ levels in males and females.

### 4.1. Cadmium

Cadmium levels in urine were the highest in subjects in Winston-Salem among all study sites. Smoking, and secondhand smoke in particular, might explain the difference in cadmium exposure in MESA communities. A previous study in NHANES showed that urinary cadmium was approximately twice and 1.5 times as high in current and former smokers as in never smokers [14]. In 2000, state-specific prevalence of current cigarette smoking among adults was similar in Illinois, Maryland, Minnesota and New York (range 19.8%–22.3%) and they ranked in the bottom half among all states [15]. Prevalence of current cigarette smoking among adults was low in California (17.2%) due to its decade-long strict smoke-free laws in public places [15,25], and markedly high in North Carolina (26.1%) [15]. Furthermore, for participants in Winston-Salem, the high prevalence of active smoking would result in an increase in secondhand smoke exposures, especially in never smokers and former smokers. In a model with further adjustment for secondhand smoke, GM decreased by 26.2% in participants in Winston-Salem. Although food is a major source of exposure to cadmium in non-smokers [12], our results remained similar after adjustment for intake of high-cadmium food groups.

### 4.2. Tungsten and Uranium

Tungsten and uranium levels in urine were the highest in participants in Los Angeles among all study sites. Drinking water from groundwater sources can be a relevant source of tungsten and uranium exposure because locations with natural formations have elevated levels of these metals in groundwater, and groundwater is a common source of water in the Western US [11,13]. California is the state with the third highest uranium concentrations in drinking water, with uranium levels of 2.7 pCi/L [16]. Although tungsten levels in drinking water are generally unknown, releases to groundwater typically occur in regions where natural formations of tungsten minerals are prevalent, including California [38].

Although human exposures to tungsten from air are very low, entry into the air occurs during tungsten ore processing, alloy fabrication, tungsten carbide production and use, as well as during municipal waste combustion [11]. This study provides cross-sectional evidence suggesting the association of PM_2.5_ level with urinary tungsten. The association is possible because: (1) the GM of urinary tungsten in participants in Los Angeles decreased by 36.4% with further adjustment for PM_2.5_ levels; and (2) the associations of PM_2.5_ tertiles with urinary tungsten persisted in models with adjustment for study sites. Tungsten is thrombogenic and proinflammatory and has been linked to cardiovascular disease, peripheral arterial disease, and possibly diabetes [2,6,7]. Our finding of differential tungsten exposure by geographical location as well as the relationship between PM_2.5_ and urinary tungsten requires additional research, as so little is known regarding the sources of tungsten for the general population, and it may guide regulations of tungsten exposure from water and air.

Uranium levels in urine were only weakly associated with PM_2.5_ levels in the model without adjustment for study sites. However, the association disappeared with further adjustment for study sites. It is possible that the apparent association of PM_2.5_ level with urinary uranium might be due to confounding by study sites, and adjustment for study sites controlled for other environmental sources of uranium (e.g., groundwater) different across study sites.

### 4.3. Limitations and Strengths

Our study has some limitations. First, our analysis was limited by the relatively small sample size, and thus we might miss weak associations between geographical locations and some metals. Second, as in most epidemiologic studies of urinary metals we used spot urine samples, which requires adjustment for urine dilution. There are scientific debates about whether it is better to adjust for urine dilution using urine specific gravity or urine creatinine. In our study, we adjusted for urinary specific gravity because urinary creatinine is also a marker of creatinine production, and thus it is associated with age, sex, and muscle mass [39]. Specific gravity corrections could introduce less variability than urinary creatinine corrections. In a sensitivity analysis with adjustment of urinary creatinine, we found similar results. Third, the use of single measurement of urinary metals might not accurately reflect long-term body burden of metals [40]. Other limitations involve the cross-sectional design and residual confounding by socioeconomic status.

Despite these limitations, this study has several strengths. We investigated the geographical differences of urinary metals across the US and the association of household-level PM_2.5_ and NOx exposure with urinary metals in a multi-ethnic cohort of the general population. Moreover, we utilized the specialized and complex monitoring exposure models from MESA Air to predict spatially resolved estimates at each participant’s home, thus minimizing measurement error in the exposure prediction [32]. This would allow us to obtain less biased measures of association. Other important strengths of this study include high quality and standard protocols of data collection, laboratory procedures to determine urinary metals, and ability to adjust for non-air pollution sources of metal exposure including smoking and diet.

## 5. Conclusions

Urinary cadmium, tungsten and uranium concentrations differed by geographic locations in MESA communities. Higher cadmium concentrations in Winston-Salem could reflect the higher prevalence of active smoking and probably the higher secondhand smoke exposure. Higher tungsten and uranium in Los Angeles might indicate higher tungsten and uranium exposure from groundwater. There was a positive association of PM_2.5_ levels with urinary tungsten after controlling for study sites, suggesting that air pollution might explain the site difference in urinary tungsten. Higher cadmium exposure in Winston-Salem underscores the need for intensive smoking prevention campaigns in North Carolina. Given the emerging evidence of health effects of tungsten and uranium in the general population, the health impact of chronic exposure to these metals requires evaluation in the population in Los Angeles. The observed association of PM_2.5_ levels and urinary tungsten warrants further research on PM_2.5_ speciation to confirm this relationship. Our study helps identify modifiable sources of toxic metal exposure that contributes to the geographical differences across MESA communities.

## Figures and Tables

**Table 1:** Characteristics of participants by study sites.

No. of Participants	Baltimore	Chicago	Los Angeles	New York City	Saint Paul	Winston-Salem
	30	65	89	55	38	27
Sex, %						
Men	60.0	67.7	66.7	41.8	62.5	40.0
Women	40.0	32.3	33.3	58.2	37.5	60.0
Age, y, mean (SD)	60.2 (9.9)	61.8 (9.6)	62.4 (10.1)	63.4 (8.6)	58.5 (8.6)	58.6 (9.4)
Race, %						
White	50.0	23.1	16.7	27.3	37.5	50.0
Chinese American	--	53.8	38.8	--	--	--
African American	50.0	23.1	16.7	27.3	--	50.0
Hispanic	--	--	27.8	45.4	62.5	--
Education, %						
≤High School	46.7	50.8	65.2	69.1	65.8	51.9
>High School	53.3	49.2	34.8	30.9	34.2	48.1
Family income, %						
<$25,000	30.0	23.1	47.8	38.2	22.5	13.3
$25,000–50,000	23.3	21.5	26.7	30.9	37.5	23.3
$50,000–75,000	13.3	6.2	6.7	14.5	25.0	30.0
≥$75,000	33.4	49.2	18.8	16.4	15.0	33.4
BMI, kg/m^2^, mean (SD)	28.4 (4.8)	24.8 (4.6)	26.5 (4.6)	29.3 (5.5)	29.6 (5.4)	28.4 (6.3)
Smoking, %						
Never Smoker	40.0	52.3	51.1	47.3	22.5	43.3
Former Smoker	33.3	32.3	38.9	40.0	60.0	53.3
Current Smoker	26.7	15.4	10.0	12.7	17.5	3.4
Secondhand smoke, %						
0 h/week	50.0	51.8	83.3	61.2	44.1	53.9
1–10 h/week	45.5	35.7	14.1	32.7	38.2	34.6
>10 h/week	4.5	12.5	2.6	6.1	17.7	11.5
Air pollution exposure, GM (95% CI)						
PM_2.5_ concentration, µg/m^3^	15.7 (14.0, 17.4)	16.2 (13.7, 18.8)	21.3 (19.0, 23.6)	16.5 (13.3, 19.7)	12.9 (11.6, 14.2)	16.5 (15.2, 17.9)
NOx concentration, ppb	39.6 (17.0, 62.2)	42.4 (21.4, 63.5)	75.6 (28.1, 123.1)	80.9 (55.0, 106.7)	23.2 (13.6, 32.8)	24.7 (7.7, 41.7)

Abbreviations: CI, confidence interval; GM, geometric mean; NOx, nitrogen oxides; PM_2.5_, fine particulate matter.

**Table 2:** Adjusted geometric means (95% confidence interval) of urinary metals by study sites.

Study Site	Model 1 ^a^	Model 2 ^b^	Model 3 ^c^	Model 4 ^d^
**Antimony**				
Winston-Salem	0.13 (0.07, 0.22)	0.12 (0.07, 0.21)	0.12 (0.07, 0.21)	0.12 (0.06, 0.24)
Baltimore	0.08 (0.06, 0.12)	0.08 (0.06, 0.12)	0.08 (0.06, 0.13)	0.08 (0.06, 0.12)
Chicago	0.11 (0.07, 0.19)	0.12 (0.07, 0.20)	0.12 (0.07, 0.21)	0.12 (0.07, 0.20)
Los Angeles	0.10 (0.07, 0.13)	0.10 (0.07, 0.13)	0.09 (0.05, 0.16)	0.10 (0.07, 0.14)
New York City	0.11 (0.07, 0.16)	0.11 (0.07, 0.16)	0.11 (0.07, 0.16)	0.11 (0.07, 0.17)
Saint Paul	0.11 (0.07, 0.19)	0.11 (0.07, 0.19)	0.13 (0.06, 0.30)	0.11 (0.06, 0.22)
**Cadmium**				
Winston-Salem	0.79 (0.54, 1.16)	0.84 (0.57, 1.22)	0.85 (0.58, 1.25)	0.92 (0.59, 1.46)
Baltimore	0.56 (0.39, 0.80)	0.51 (0.36, 0.72)	0.54 (0.37, 0.78)	0.53 (0.37, 0.75)
Chicago	0.61 (0.47, 0.79)	0.60 (0.47, 0.78)	0.62 (0.48, 0.82)	0.61 (0.47, 0.79)
Los Angeles	0.61 (0.47, 0.79)	0.60 (0.49, 0.73)	0.51 (0.34, 0.77)	0.56 (0.44, 0.72)
New York City	0.62 (0.47, 0.81)	0.63 (0.49, 0.82)	0.65 (0.50, 0.85)	0.59 (0.43, 0.81)
Saint Paul	0.66 (0.47, 0.93)	0.61 (0.44, 0.85)	0.74 (0.42, 1.31)	0.68 (0.44, 1.07)
**Tungsten**				
Winston-Salem	0.032 (0.017, 0.061)	0.032 (0.017, 0.061)	0.034 (0.018, 0.065)	0.043 (0.020, 0.094)
Baltimore	0.031 (0.017, 0.057)	0.030 (0.017, 0.055)	0.036 (0.019, 0.068)	0.033 (0.018, 0.061)
Chicago	0.032 (0.021, 0.050)	0.033 (0.021, 0.050)	0.036 (0.023, 0.057)	0.034 (0.022, 0.053)
Los Angeles	0.11 (0.078, 0.16)	0.11 (0.079, 0.16)	0.070 (0.035, 0.14)	0.096 (0.063, 0.15)
New York City	0.033 (0.021, 0.051)	0.033 (0.021, 0.052)	0.036 (0.023, 0.057)	0.027 (0.016, 0.047)
Saint Paul	0.026 (0.015, 0.046)	0.025 (0.014, 0.044)	0.047 (0.018, 0.12)	0.035 (0.016, 0.074)
**Uranium**				
Winston-Salem	0.013 (0.009, 0.018)	0.013 (0.009, 0.018)	0.013 (0.010, 0.018)	0.014 (0.010, 0.021)
Baltimore	0.012 (0.009, 0.016)	0.012 (0.009, 0.016)	0.012 (0.009, 0.017)	0.012 (0.009, 0.017)
Chicago	0.012 (0.009, 0.014)	0.012 (0.009, 0.014)	0.012 (0.009, 0.015)	0.012 (0.009, 0.015)
Los Angeles	0.019 (0.016, 0.023)	0.019 (0.016, 0.023)	0.019 (0.013, 0.026)	0.018 (0.015, 0.023)
New York City	0.011 (0.009, 0.013)	0.011 (0.009, 0.013)	0.011 (0.009, 0.014)	0.010 (0.008, 0.013)
Saint Paul	0.009 (0.006, 0.012)	0.009 (0.007, 0.012)	0.009 (0.006, 0.015)	0.010 (0.007, 0.014)

**^a^** Adjusted for age, sex, race/ethnicity, BMI, education, and annual family income; **^b^** Adjusted for variables in Model 1, plus smoking status and pack-year; **^c^** Adjusted for variables in Model 2, plus log-transformed PM_2.5_; **^d^** Adjusted for variables in Model 2, plus log-transformed NOx.

**Table 3:** Ratios of geometric means (95% confidence interval) of urinary metals by tertiles of PM_2.5_ concentrations **^a^**.

PM_2.5_	*N*	Model 1 ^b^	Model 2 ^c^	Model 3 ^d^	Model 4 ^e^
**Antimony**					
<15.7 µg/m^3^	101	1.00 (Reference)	1.00 (Reference)	1.00 (Reference)	1.00 (Reference)
15.7–18.3 µg/m^3^	101	1.08 (0.73, 1.60)	1.07 (0.73, 1.58)	1.09 (0.72, 1.64)	1.14 (0.72, 1.80)
≥18.3 µg/m^3^	102	0.93 (0.62, 1.38)	0.92 (0.62, 1.38)	0.94 (0.58, 1.54)	0.68 (0.28, 1.64)
Per 5 µg/m^3^^f^	304	0.98 (0.74, 1.29)	0.97 (0.73, 1.28)	1.00 (0.69, 1.46)	1.03 (0.51, 2.08)
*p* for trend		0.61	0.6	0.69	0.51
**Cadmium**					
<15.7 µg/m^3^	101	1.00 (Reference)	1.00 (Reference)	1.00 (Reference)	1.00 (Reference)
15.7–18.3 µg/m^3^	101	1.00 (0.77, 1.30)	1.00 (0.78, 1.28)	1.03 (0.79, 1.35)	0.93 (0.69, 1.25)
≥18.3 µg/m^3^	102	1.01 (0.77, 1.32)	1.08 (0.83, 1.40)	1.15 (0.84, 1.58)	1.43 (0.81, 2.52)
Per 5 µg/m^3^	304	0.97 (0.80, 1.17)	1.03 (0.86, 1.23)	1.08 (0.85, 1.38)	1.08 (0.69, 1.70)
*p* for trend		0.95	0.54	0.35	0.32
**Tungsten**					
<15.7 µg/m^3^	101	1.00 (Reference)	1.00 (Reference)	1.00 (Reference)	1.00 (Reference)
15.7–18.3 µg/m^3^	101	**1.64 (1.05, 2.56)**	**1.64 (1.05, 2.56)**	1.57 (0.98, 2.52)	**1.71 (1.02, 2.88)**
≥18.3 µg/m^3^	102	**3.47 (2.20, 5.49)**	**3.55 (2.24, 5.63)**	**3.25 (1.86, 5.69)**	0.77 (0.29, 2.09)
Per 5 µg/m^3^	304	**2.53 (1.84, 3.48)**	**2.59 (1.88, 3.56)**	**2.74 (1.79, 4.18)**	1.57 (0.71, 3.48)
*p* for trend		<0.001	<0.001	<0.001	0.99
**Uranium**					
<15.7 µg/m^3^	101	1.00 (Reference)	1.00 (Reference)	1.00 (Reference)	1.00 (Reference)
15.7–18.3 µg/m^3^	101	1.18 (0.94, 1.47)	1.18 (0.94, 1.48)	1.17 (0.92, 1.49)	1.08 (0.82, 1.40)
≥18.3 µg/m^3^	102	**1.71 (1.36, 2.16)**	**1.70 (1.34, 2.14)**	**1.69 (1.27, 2.25)**	0.84 (0.51, 1.40)
Per 5 µg/m^3^	304	**1.51 (1.29, 1.78)**	**1.50 (1.28, 1.77)**	**1.61 (1.29, 1.99)**	0.98 (0.66, 1.47)
*p* for trend		<0.001	<0.001	<0.001	0.63

**^a^** Results are in bold where it reached statistical significance (α = 0.05) in a model. Ratios of geometric means are equivalent to antilogarithm (exponentiation) of regression coefficients; **^b^** Adjusted for age, sex, race/ethnicity, BMI, education, and annual family income; **^c^** Adjusted for variables in Model 1, plus smoking status and pack-year; **^d^** Adjusted for variables in Model 2, plus log-transformed NOx; **^e^** Adjusted for variables in Model 2, plus study site; ^f^ Interquartile range for PM_2.5_.

## Abbreviations

The following abbreviations are used in this manuscript:

BMI	Body Mass Index
Egfr	Estimated Glomerular Filtration Rate
GM	Geometric Mean
LOD	Limit of Detection
NOx	Oxides of Nitrogen
PM_2.5_	Fine Particulate Matter

## Appendix

**Table A1:** Ratios of geometric means (95% confidence interval) of urinary cadmium by study sites ^a^.

Cadmium	Model 1 ^b^	Model 2 ^c^
Winston-Salem	1.00 (Reference)	1.00 (Reference)
Baltimore	0.71 (0.29,1.77)	**0.61 (0.38,0.99)**
Chicago	0.80 (0.36,1.79)	0.82 (0.52,1.30)
Los Angeles	0.90 (0.41,1.98)	0.81 (0.52,1.26)
New York City	0.91 (0.41,1.98)	0.73 (0.47,1.15)
Saint Paul	0.88 (0.33,2.35)	0.80 (0.49,1.32)

^a^ Results are in bold where it reached statistical significance (α = 0.05) in a model. ^b^ Adjusted for age, sex, race/ethnicity, BMI, education, annual family income, smoking status, pack-year and secondhand smoke. ^c^ Adjusted for age, sex, race/ethnicity, BMI, education, annual family income, smoking status, pack-year and high-Cd food (fruits, vegetables, grains, nuts, red meat and shellfish).

**Table A2:** Adjusted geometric means (95% confidence interval) of urinary metals by study sites.

Study Site	Model 1 ^a^	Model 2 ^b^	Model 3 ^c^	Model 4 ^d^
**Antimony**				
Winston-Salem	0.13 (0.07, 0.22)	0.12 (0.07, 0.21)	0.12 (0.07, 0.21)	0.12 (0.06, 0.24)
Baltimore	0.08 (0.06, 0.12)	0.08 (0.06, 0.12)	0.09 (0.06, 0.13)	0.08 (0.06, 0.12)
Chicago	0.11 (0.07, 0.19)	0.12 (0.07, 0.20)	0.12 (0.07, 0.21)	0.12 (0.07, 0.20)
Los Angeles	0.10 (0.07, 0.13)	0.10 (0.07, 0.13)	0.09 (0.05, 0.16)	0.10 (0.07, 0.14)
New York City	0.11 (0.07, 0.16)	0.11 (0.07, 0.16)	0.11 (0.07, 0.16)	0.11 (0.07, 0.17)
Saint Paul	0.11 (0.07, 0.19)	0.11 (0.07, 0.19)	0.13 (0.05, 0.30)	0.11 (0.06, 0.22)
**Cadmium**				
Winston-Salem	0.80 (0.54, 1.17)	0.84 (0.58, 1.23)	0.86 (0.59, 1.26)	0.94 (0.59, 1.49)
Baltimore	0.57 (0.39, 0.81)	0.52 (0.36, 0.74)	0.55 (0.38, 0.80)	0.53 (0.37, 0.76)
Chicago	0.62 (0.47, 0.80)	0.61 (0.47, 0.79)	0.63 (0.48, 0.83)	0.62 (0.48, 0.80)
Los Angeles	0.57 (0.46, 0.70)	0.59 (0.49, 0.73)	0.51 (0.34, 0.76)	0.56 (0.44, 0.72)
New York City	0.62 (0.47, 0.80)	0.63 (0.48, 0.82)	0.64 (0.50, 0.84)	0.58 (0.42, 0.80)
Saint Paul	0.64 (0.45, 0.91)	0.59 (0.43, 0.84)	0.74 (0.42, 1.30)	0.67 (0.43, 1.05)
**Tungsten**				
Winston-Salem	0.034 (0.018, 0.063)	0.033 (0.018, 0.064)	0.036 (0.019, 0.069)	0.046 (0.021, 0.099)
Baltimore	0.033 (0.018, 0.059)	0.032 (0.017, 0.058)	0.039 (0.020, 0.073)	0.035 (0.019, 0.067)
Chicago	0.034 (0.022, 0.052)	0.034 (0.022, 0.052)	0.038 (0.024, 0.060)	0.035 (0.023, 0.056)
Los Angeles	0.11 (0.077, 0.15)	0.11 (0.08, 0.16)	0.066 (0.033, 0.13)	0.093 (0.061, 0.14)
New York City	0.032 (0.021, 0.050)	0.033 (0.021, 0.051)	0.036 (0.023, 0.056)	0.033 (0.015, 0.045)
Saint Paul	0.024 (0.014, 0.042)	0.023 (0.013, 0.041)	0.046 (0.018, 0.12)	0.033 (0.015, 0.071)
**Uranium**				
Winston-Salem	0.013 (0.009, 0.018)	0.013 (0.010, 0.018)	0.013 (0.010, 0.019)	0.014 (0.010, 0.021)
Baltimore	0.012 (0.009, 0.016)	0.012 (0.009, 0.017)	0.012 (0.009, 0.017)	0.013 (0.009, 0.017)
Chicago	0.012 (0.009, 0.015)	0.012 (0.009, 0.015)	0.012 (0.009, 0.015)	0.012 (0.009, 0.015)
Los Angeles	0.019 (0.016, 0.023)	0.019 (0.016, 0.023)	0.018 (0.011, 0.026)	0.018 (0.015, 0.023)
New York City	0.011 (0.009, 0.013)	0.011 (0.008, 0.013)	0.011 (0.009, 0.013)	0.010 (0.008, 0.013)
Saint Paul	0.008 (0.006, 0.011)	0.009 (0.006, 0.012)	0.009 (0.006, 0.015)	0.010 (0.006, 0.014)

**^a^** Adjusted for age, sex, race/ethnicity, BMI, education, annual family income, and estimated glomerular filtration rate (eGFR); **^b^** Adjusted for variables in Model 1, plus smoking status and pack-year; **^c^** Adjusted for variables in Model 2, plus log-transformed PM_2.5_; **^d^** Adjusted for variables in Model 2, plus log-transformed NOx.

**Table A3:** Ratios of geometric means (95% confidence interval) of urinary metals by tertiles of PM_2.5_ concentrations ^a^.

PM_2.5_	*N*	Model 1 ^b^	Model 2 ^c^	Model 3 ^d^	Model 4 ^e^
**Antimony**					
<15.7 µg/m^3^	101	1.00 (Reference)	1.00 (Reference)	1.00 (Reference)	1.00 (Reference)
15.7–18.3 µg/m^3^	101	0.93 (0.62, 1.39)	0.92 (0.62, 1.38)	0.94 (0.58, 1.54)	0.68 (0.28, 1.66)
≥18.3 µg/m^3^	102	0.98 (0.74, 1.29)	0.97 (0.73, 1.28)	1.00 (0.69, 1.46)	1.03 (0.51, 2.08)
Per 5 µg/m^3^^f^	304	1.09 (0.74, 1.60)	1.08 (0.73, 1.59)	1.09 (0.72, 1.65)	1.14 (0.72, 1.81)
*p* for trend		0.63	0.63	0.61	0.69
**Cadmium**					
<15.7 µg/m^3^	101	1.00 (Reference)	1.00 (Reference)	1.00 (Reference)	1.00 (Reference)
15.7–18.3 µg/m^3^	101	1.01 (0.77, 1.33)	1.08 (0.83, 1.40)	1.15 (0.84, 1.58)	1.47 (0.83, 2.61)
≥18.3 µg/m^3^	102	0.97 (0.80, 1.17)	1.03 (0.86, 1.23)	1.08 (0.85, 1.38)	1.08 (0.69, 1.70)
Per 5 µg/m^3^	304	1.01 (0.77, 1.31)	1.01 (0.78, 1.30)	1.04 (0.79, 1.36)	0.93 (0.69, 1.25)
*p* for trend		0.95	0.54	0.37	0.29
**Tungsten**					
<15.7 µg/m^3^	101	1.00 (Reference)	1.00 (Reference)	1.00 (Reference)	1.00 (Reference)
15.7–18.3 µg/m^3^	101	**1.70 (1.09, 2.66)**	**1.70 (1.09, 2.66)**	**1.63 (1.02, 2.61)**	**1.72 (1.02, 2.89)**
≥18.3 µg/m^3^	102	**3.54 (2.24, 5.58)**	**3.60 (2.27, 5.69)**	**3.28 (1.88, 5.73)**	0.85 (0.31, 2.32)
Per 5 µg/m^3^	304	**2.53 (1.84, 3.48)**	**2.59 (1.88, 3.56)**	**2.74 (1.79, 4.18)**	1.57 (0.71, 3.48)
*p* for trend		<0.001	<0.001	<0.001	0.90
**Uranium**					
<15.7 µg/m^3^	101	1.00 (Reference)	1.00 (Reference)	1.00 (Reference)	1.00 (Reference)
15.7–18.3 µg/m^3^	101	1.18 (0.94, 1.48)	1.18 (0.94, 1.48)	1.18 (0.93, 1.50)	1.08 (0.82, 1.40)
≥18.3 µg/m^3^	102	**1.72 (1.36, 2.17)**	**1.70 (1.34, 2.15)**	**1.69 (1.27, 2.25)**	0.85 (0.51, 1.42)
Per 5 µg/m^3^	304	**1.51 (1.29, 1.78)**	**1.50 (1.28, 1.77)**	**1.61 (1.29, 1.99)**	0.98 (0.66, 1.47)
*p* for trend		<0.001	<0.001	<0.001	0.76

^a^ Results are in bold where it reached statistical significance (α = 0.05) in a model; **^b^** Adjusted for age, sex, race/ethnicity, BMI, education, annual family income, and estimated glomerular filtration rate (eGFR); **^c^** Adjusted for variables in Model 1, plus smoking status and pack-year; **^d^** Adjusted for variables in Model 2, plus log-transformed NOx; **^e^** Adjusted for variables in Model 2, plus study site; ^f^ Interquartile range for PM_2.5_.

**Table A4:** Ratios of geometric means (95% confidence interval) of urinary metals by tertiles of NOx concentrations ^a^.

NOx	*N*	Model 1 ^b^	Model 2 ^c^	Model 3 ^d^	Model 4 ^e^
**Antimony**					
<37.9 ppb	101	1.00 (Reference)	1.00 (Reference)	1.00 (Reference)	1.00 (Reference)
37.9–69.0 ppb	101	0.95 (0.63, 1.43)	0.97 (0.64, 1.46)	1.00 (0.64, 1.56)	1.19 (0.69, 2.04)
≥69.0 ppb	102	1.04 (0.68, 1.59)	1.05 (0.68, 1.62)	1.11 (0.64, 1.93)	1.36 (0.66, 2.80)
Per 45 ppb ^f^	304	0.93 (0.76, 1.13)	0.98 (0.81, 1.19)	0.93 (0.72, 1.21)	1.06 (0.77, 1.46)
*p* for trend		0.38	0.74	0.52	0.93
**Cadmium**					
<37.9 ppb	101	1.00 (Reference)	1.00 (Reference)	1.00 (Reference)	1.00 (Reference)
37.9–69.0 ppb	101	0.82 (0.62, 1.08)	0.82 (0.63, 1.07)	0.78 (0.58, 1.04)	0.83 (0.59, 1.19)
≥69.0 ppb	102	0.86 (0.64, 1.15)	0.92 (0.70, 1.22)	0.84 (0.59, 1.19)	0.94 (0.59, 1.49)
Per 45 ppb	304	0.93 (0.76, 1.13)	0.98 (0.81, 1.19)	0.93 (0.72, 1.21)	1.06 (0.77, 1.46)
*p* for trend		0.80	0.77	0.65	0.42
**Tungsten**					
<37.9 ppb	101	1.00 (Reference)	1.00 (Reference)	1.00 (Reference)	1.00 (Reference)
37.9–69.0 ppb	101	1.51 (0.94, 2.42)	1.51 (0.94, 2.44)	0.95 (0.57, 1.58)	1.03 (0.76, 1.41)
≥69.0 ppb	102	**2.63 (1.59, 4.33)**	**2.69 (1.62, 4.46)**	1.13 (0.61, 2.10)	1.05 (0.69, 1.59)
Per 45 ppb	304	**1.84 (1.31, 2.59)**	**1.87 (1.32, 2.65)**	0.90 (0.57, 1.43)	1.37 (0.78, 2.40)
*p* for trend		<0.001	<0.001	0.64	0.04
**Uranium**					
<37.9 ppb	101	1.00 (Reference)	1.00 (Reference)	1.00 (Reference)	1.00 (Reference)
37.9–69.0 ppb	101	1.15 (0.90, 1.47)	1.15 (0.90, 1.47)	0.90 (0.70, 1.17)	1.03 (0.76, 1.41)
≥69.0 ppb	102	**1.33 (1.03, 1.72)**	**1.31 (1.01, 1.70)**	0.84 (0.61, 1.15)	1.05 (0.69, 1.59)
Per 45 ppb	304	**1.28 (1.07, 1.52)**	**1.26 (1.06, 1.51)**	0.90 (0.71, 1.14)	1.10 (0.82, 1.46)
*p* for trend		0.026	0.038	0.30	0.68

^a^ Results are in bold where it reached statistical significance (α = 0.05) in a model; **^b^** Adjusted for age, sex, race/ethnicity, BMI, education, annual family income, and estimated glomerular filtration rate (eGFR); **^c^** Adjusted for variables in Model 1, plus smoking status and pack-year; **^d^** Adjusted for variables in Model 2, plus log-transformed PM_2.5_; **^e^** Adjusted for variables in Model 2, plus study site; ^f^ Interquartile range for NOx.

**Table A5:** Adjusted geometric means (95% confidence interval) of urinary metals by study sites.

Study site	Model 1 ^a^	Model 2 ^b^	Model 3 ^c^	Model 4 ^d^
**Tungsten**				
Winston-Salem	0.067 (0.044, 0.10)	0.065 (0.042, 0.10)	0.068 (0.044, 0.10)	0.097 (0.058, 0.16)
Baltimore	0.058 (0.039, 0.086)	0.057 (0.038, 0.085)	0.065 (0.042, 0.098)	0.064 (0.043, 0.095)
Chicago	0.080 (0.060, 0.11)	0.081 (0.061, 0.11)	0.088 (0.065, 0.12)	0.086 (0.065, 0.12)
Los Angeles	0.15 (0.12, 0.19)	0.15 (0.12, 0.19)	0.11 (0.068, 0.17)	0.12 (0.093, 0.16)
New York City	0.071 (0.053, 0.096)	0.073 (0.054, 0.097)	0.077 (0.057, 0.10)	0.054 (0.038, 0.077)
Saint Paul	0.11 (0.073, 0.15)	0.10 (0.069, 0.15)	0.16 (0.085, 0.30)	0.16 (0.096, 0.26)
**Uranium**				
Winston-Salem	0.021 (0.016, 0.028)	0.022 (0.017, 0.030)	0.022 (0.017, 0.030)	0.024 (0.017, 0.034)
Baltimore	0.018 (0.014, 0.024)	0.018 (0.014, 0.024)	0.018 (0.013, 0.024)	0.018 (0.014, 0.024)
Chicago	0.017 (0.014, 0.021)	0.017 (0.014, 0.020)	0.017 (0.014, 0.020)	0.017 (0.014, 0.021)
Los Angeles	0.030 (0.025, 0.034)	0.030 (0.025, 0.034)	0.031 (0.023, 0.042)	0.028 (0.024, 0.034)
New York City	0.015 (0.013, 0.018)	0.015 (0.013, 0.018)	0.015 (0.012, 0.018)	0.014 (0.011, 0.018)
Saint Paul	0.020 (0.015, 0.025)	0.020 (0.015, 0.025)	0.019 (0.012, 0.028)	0.021 (0.015, 0.030)

^a^ Adjusted for age, sex, race/ethnicity, BMI, education, and annual family income; ^b^ Adjusted for variables in Model 1, plus smoking status and pack-year; ^c^ Adjusted for variables in Model 2, plus log-transformed PM_2.5_; ^d^ Adjusted for variables in Model 2, plus log-transformed NOx.

**Table A6:** Ratios of geometric means (95% confidence interval) of urinary metals by tertiles of PM_2.5_ concentrations ^a^.

PM_2.5_	*N*	Model 1 ^b^	Model 2 ^c^	Model 3 ^d^	Model 4 ^e^
**Tungsten**					
<15.7 µg/m^3^	101	1.00 (Reference)	1.00 (Reference)	1.00 (Reference)	1.00 (Reference)
15.7–18.3 µg/m^3^	101	1.16 (0.79, 1.70)	1.16 (0.79, 1.70)	1.08 (0.71, 1.65)	1.54 (0.99, 2.39)
≥18.3 µg/m^3^	102	**2.50 (1.70, 3.67)**	**2.58 (1.75, 3.80)**	**2.24 (1.39, 3.60)**	1.74 (0.68, 4.40)
Per 5 µg/m^3^^f^	304	**1.87 (1.40, 2.51)**	**1.94 (1.44, 2.61)**	**1.74 (1.20, 2.54)**	1.63 (0.78, 3.44)
*p* for trend		<0.001	<0.001	<0.001	0.12
**Uranium**					
<15.7 µg/m^3^	101	1.00 (Reference)	1.00 (Reference)	1.00 (Reference)	1.00 (Reference)
15.7–18.3 µg/m^3^	101	0.95 (0.72, 1.25)	0.95 (0.72, 1.26)	0.99 (0.73, 1.34)	0.98 (0.73, 1.31)
≥18.3 µg/m^3^	102	**1.51 (1.11, 2.06)**	**1.52 (1.11, 2.08)**	**1.64 (1.12, 2.40)**	0.74 (0.41, 1.31)
Per 5 µg/m^3^	304	**1.64 (1.33, 2.01)**	**1.67 (1.36, 2.05)**	**1.80 (1.39, 2.32)**	0.85 (0.52, 1.37)
*p* for trend		<0.001	<0.001	<0.001	0.43

^a^ Results are in bold where it reached statistical significance (α = 0.05) in a model; **^b^** Adjusted for age, sex, race/ethnicity, BMI, education, and annual family income; **^c^** Adjusted for variables in Model 1, plus smoking status and pack-year; ^d^ Adjusted for variables in Model 2, plus log-transformed NOx; **^e^** Adjusted for variables in Model 2, plus study site; ^f^ Interquartile range for PM_2.5_.

**Table A7:** Ratios of geometric means (95% confidence interval) of urinary metals by tertiles of NOx concentrations ^a^.

NOx	*N*	Model 1 ^b^	Model 2 ^c^	Model 3 ^d^	Model 4 ^e^
**Tungsten**					
<37.9 ppb	101	1.00 (Reference)	1.00 (Reference)	1.00 (Reference)	1.00 (Reference)
37.9–69.0 ppb	101	1.11 (0.75, 1.63)	1.11 (0.76, 1.64)	0.88 (0.57, 1.35)	1.14 (0.69, 1.89)
≥69.0 ppb	102	**2.02 (1.33, 3.06)**	**2.10 (1.38, 3.21)**	1.35 (0.78, 2.32)	1.73 (0.85, 3.53)
Per 45 ppb ^f^	304	**1.79 (1.31, 2.45)**	**1.85 (1.35, 2.55)**	1.34 (0.88, 2.03)	1.77 (1.08, 2.88)
*p* for trend		0.002	0.001	0.23	0.05
**Uranium**					
<37.9 ppb	101	1.00 (Reference)	1.00 (Reference)	1.00 (Reference)	1.00 (Reference)
37.9–69.0 ppb	101	0.37 (0.65, 1.26)	0.89 (0.64, 1.23)	0.73 (0.51, 1.04)	0.88 (0.61, 1.25)
≥69.0 ppb	102	1.18 (0.88, 1.60)	1.17 (0.86, 1.59)	0.82 (0.56, 1.20)	1.01 (0.65, 1.55)
Per 45 ppb	304	**1.37 (1.09, 1.73)**	**1.38 (1.10, 1.74)**	0.99 (0.74, 1.31)	1.05 (0.76, 1.45)
*p* for trend		0.015	0.016	0.97	0.62

^a^ Results are in bold where it reached statistical significance (α = 0.05) in a model; **^b^** Adjusted for age, sex, race/ethnicity, BMI, education, and annual family income; **^c^** Adjusted for variables in Model 1, plus smoking status and pack-year; ^d^ Adjusted for variables in Model 2, plus log-transformed PM_2.5_; **^e^** Adjusted for variables in Model 2, plus study site; ^f^ Interquartile range for NOx.

**Table A8:** Adjusted geometric means (95% confidence interval) of urinary metals by study sites in males.

Study Site	Model 1 ^a^	Model 2 ^b^	Model 3 ^c^	Model 4 ^d^
**Antimony**				
Winston-Salem	0.11 (0.05, 0.25)	0.10 (0.043, 0.25)	0.11 (0.05, 0.26)	0.15 (0.05, 0.38)
Baltimore	0.06 (0.03, 0.15)	0.06 (0.03, 0.14)	0.07 (0.03, 0.17)	0.06 (0.03, 0.15)
Chicago	0.09 (0.05, 0.15)	0.09 (0.05, 0.15)	0.10 (0.06, 0.18)	0.09 (0.06, 0.16)
Los Angeles	0.11 (0.08, 0.16)	0.11 (0.08, 0.17)	0.07 (0.03, 0.17)	0.09 (0.06, 0.15)
New York City	0.09 (0.05, 0.18)	0.10 (0.05, 0.18)	0.11 (0.06, 0.21)	0.07 (0.04, 0.15)
Saint Paul	0.10 (0.05, 0.20)	0.10 (0.05, 0.19)	0.20 (0.05, 0.75)	0.15 (0.06, 0.37)
**Cadmium**				
Winston-Salem	0.73 (0.40, 1.31)	0.72 (0.41, 1.28)	0.74 (0.41, 1.31)	0.75 (0.39, 1.42)
Baltimore	0.54 (0.30, 0.98)	0.46 (0.26, 0.81)	0.49 (0.27, 0.87)	0.46 (0.26, 0.81)
Chicago	0.56 (0.40, 0.79)	0.54 (0.39, 0.76)	0.58 (0.40, 0.82)	0.55 (0.39, 0.76)
Los Angeles	0.48 (0.37, 0.63)	0.51 (0.04, 0.66)	0.42 (0.24, 0.75)	0.50 (0.37, 0.68)
New York City	0.45 (0.29, 0.68)	0.47 (0.32, 0.71)	0.50 (0.32, 0.76)	0.46 (0.29, 0.73)
Saint Paul	0.57 (0.35, 0.91)	0.53 (0.34, 0.84)	0.71 (0.30, 1.70)	0.56 (0.31, 1.01)
**Tungsten**				
Winston-Salem	0.046 (0.018, 0.12)	0.047 (0.018, 0.12)	0.050 (0.019, 0.13)	0.062 (0.021, 0.18)
Baltimore	0.030 (0.012, 0.077)	0.029 (0.011, 0.073)	0.033 (0.013, 0.088)	0.029 (0.011, 0.076)
Chicago	0.033 (0.019, 0.058)	0.033 (0.019, 0.057)	0.038 (0.021, 0.069)	0.035 (0.020, 0.060)
Los Angeles	0.11 (0.074, 0.17)	0.11 (0.075, 0.17)	0.069 (0.027, 0.18)	0.097 (0.059, 0.16)
New York City	0.027 (0.014, 0.052)	0.027 (0.014, 0.11)	0.031 (0.015, 0.063)	0.022 (0.010, 0.047)
Saint Paul	0.018 (0.008, 0.038)	0.017 (0.008, 0.037)	0.037 (0.0090.16)	0.025 (0.009, 0.067)
**Uranium**				
Winston-Salem	0.013 (0.008, 0.021)	0.013 (0.008, 0.022)	0.013 (0.008, 0.022)	0.013 (0.008, 0.024)
Baltimore	0.011 (0.007, 0.019)	0.011 (0.007, 0.019)	0.011 (0.007, 0.019)	0.011 (0.007, 0.019)
Chicago	0.011 (0.009, 0.015)	0.012 (0.009, 0.015)	0.011 (0.008, 0.015)	0.011 (0.009, 0.015)
Los Angeles	0.018 (0.015, 0.022)	0.018 (0.014, 0.022)	0.020 (0.012, 0.033)	0.018 (0.014, 0.023)
New York City	0.011 (0.008, 0.016)	0.011 (0.008, 0.016)	0.011 (0.008, 0.016)	0.011 (0.007, 0.017)
Saint Paul	0.007 (0.005, 0.017)	0.007 (0.005, 0.011)	0.006 (0.003, 0.014)	0.008 (0.005, 0.013)

^a^ Adjusted for age, race/ethnicity, BMI, education, and annual family income; **^b^** Adjusted for variables in Model 1, plus smoking status and pack-year; **^c^** Adjusted for variables in Model 2, plus log-transformed PM_2.5_; **^d^** Adjusted for variables in Model 2, plus log-transformed NOx.

**Table A9:** Adjusted geometric means (95% confidence interval) of urinary metals by study sites in females.

Study site	Model 1 ^a^	Model 2 ^b^	Model 3 ^c^	Model 4 ^d^
**Antimony**				
Winston-Salem	0.12 (0.06, 0.26)	0.11 (0.05, 0.24)	0.11 (0.05, 0.24)	0.06 (0.02, 0.17)
Baltimore	0.07 (0.04, 0.15)	0.07 (0.04, 0.14)	0.07 (0.04, 0.14)	0.07 (0.03, 0.13)
Chicago	0.15 (0.07, 0.29)	0.15 (0.08, 0.30)	0.14 (0.07, 0.29)	0.12 (0.06, 0.25)
Los Angeles	0.08 (0.05, 0.14)	0.08 (0.04, 0.14)	0.10 (0.04, 0.28)	0.11 (0.06, 0.24)
New York City	0.12 (0.07, 0.20)	0.12 (0.07, 0.21)	0.12 (0.07, 0.20)	0.19 (0.09, 0.41)
Saint Paul	0.14 (0.06, 0.32)	0.15 (0.07, 0.36)	0.11 (0.03, 0.40)	0.08 (0.02, 0.24)
**Cadmium**				
Winston-Salem	0.84 (0.50, 1.39)	0.91 (0.56, 1.48)	0.88 (0.54, 1.43)	0.79 (0.39, 1.60)
Baltimore	0.57 (0.36, 0.90)	0.54 (0.35, 0.83)	0.48 (0.30, 0.77)	0.51 (0.32, 0.82)
Chicago	0.63 (0.40, 0.99)	0.68 (0.44, 1.05)	0.66 (0.43, 1.02)	0.67 (0.43, 1.04)
Los Angeles	0.78 (0.54, 0.89)	0.82 (0.58, 1.17)	1.11 (0.59, 2.09)	0.89 (0.56, 1.42)
New York City	0.82 (0.58, 1.16)	0.80 (0.58, 1.11)	0.78 (0.56, 1.08)	0.88 (0.55, 1.41)
Saint Paul	0.99 (0.58, 1.71)	0.81 (0.48, 1.39)	0.58 (0.26, 1.29)	0.71 (0.33, 1.50)
**Tungsten**				
Winston-Salem	0.022 (0.009, 0.054)	0.023 (0.009, 0.057)	0.024 (0.009, 0.060)	0.024 (0.006, 0.089)
Baltimore	0.028 (0.013, 0.062)	0.028 (0.012, 0.062)	0.031 (0.013, 0.075)	0.028 (0.011, 0.069)
Chicago	0.034 (0.015, 0.076)	0.035 (0.016, 0.079)	0.036 (.016, 0.082)	0.035 (0.015, 0.080)
Los Angeles	0.13 (0.007, 0.25)	0.13 (0.07, 0.25)	0.098 (0.030, 0.33)	0.13 (0.005, 0.30)
New York City	0.035 (0.019, 0.065)	0.035 (0.019, 0.065)	0.036 (0.019, 0.067)	0.034 (0.014, 0.083)
Saint Paul	0.045 (0.017, 0.12)	0.043 (0.016, 0.12)	0.060 (0.013, 0.27)	0.045 (0.011, 0.19)
**Uranium**				
Winston-Salem	0.014 (0.009, 0.022)	0.014 (0.009, 0.021)	0.014 (0.009, 0.022)	0.015 (0.008, 0.030)
Baltimore	0.012 (0.008, 0.018)	0.012 (0.008, 0.018)	0.012 (0.008, 0.019)	0.013 (0.008, 0.020)
Chicago	0.013 (0.009, 0.020)	0.013 (0.009, 0.020)	0.013 (0.009, 0.020)	0.013 (0.009, 0.020)
Los Angeles	0.023 (0.017, 0.032)	0.023 (0.017, 0.032)	0.023 (0.013, 0.041)	0.021 (0.014, 0.033)
New York City	0.010 (0.007, 0.014)	0.010 (0.008, 0.014)	0.010 (0.007, 0.014)	0.009 (0.006, 0.014)
Saint Paul	0.009 (0.005, 0.014)	0.009 (0.005, 0.015)	0.009 (0.004, 0.019)	0.010 (0.005, 0.020)

^a^ Adjusted for age, race/ethnicity, BMI, education, and annual family income; **^b^** Adjusted for variables in Model 1, plus smoking status and pack-year; **^c^** Adjusted for variables in Model 2, plus log-transformed PM_2.5_; **^d^** Adjusted for variables in Model 2, plus log-transformed NOx.

**Table A10:** Ratios of geometric means (95% confidence interval) of urinary metals by tertiles of PM_2.5_ concentrations in males ^a^.

PM_2.5_	*N*	Model 1 ^b^	Model 2 ^c^	Model 3 ^d^	Model 4 ^e^
**Antimony**					
<15.7 µg/m^3^	58	1.00 (Reference)	1.00 (Reference)	1.00 (Reference)	1.00 (Reference)
15.7–18.3 µg/m^3^	52	1.45 (0.83, 2.52)	1.45 (0.83, 2.52)	1.34 (0.74, 2.42)	1.56 (0.81, 3.03)
≥18.3 µg/m^3^	64	1.28 (0.75, 2.19)	1.29 (0.75, 2.21)	1.11 (0.58, 2.15)	0.36 (0.08, 1.53)
Per 5 µg/m^3^^f^	174	1.24 (0.86, 1.78)	1.25 (0.86, 1.80)	1.14 (0.69, 1.89)	1.92 (0.65, 5.70)
*p* for trend		0.56	0.54	0.98	0.43
**Cadmium**					
<15.7 µg/m^3^	58	1.00 (Reference)	1.00 (Reference)	1.00 (Reference)	1.00 (Reference)
15.7–18.3 µg/m^3^	52	1.17 (0.80, 1.71)	1.15 (0.80, 1.65)	1.22 (0.83, 1.80)	1.15 (0.74, 1.78)
≥18.3 µg/m^3^	64	1.02 (0.70, 1.48)	1.07 (0.75, 1.53)	1.19 (0.78, 1.83)	1.48 (0.57, 3.83)
Per 5 µg/m^3^	174	0.96 (0.75, 1.23)	1.01 (0.79, 1.28)	1.10 (0.79, 1.52)	1.27 (0.62, 2.57)
*p* for trend		0.93	0.79	0.54	0.29
**Tungsten**					
<15.7 µg/m^3^	58	1.00 (Reference)	1.00 (Reference)	1.00 (Reference)	1.00 (Reference)
15.7–18.3 µg/m^3^	52	**2.07 (1.12, 3.82)**	**2.06 (1.12, 3.82)**	**1.95 (1.01, 3.76)**	1.78 (0.86, 3.66)
≥18.3 µg/m^3^	64	**4.49 (2.48, 8.16)**	**4.55 (2.49, 8.29)**	**4.10 (1.98, 8.50)**	0.46 (0.10, 2.22)
Per 5 µg/m^3^	174	**2.96 (1.99, 4.39)**	**3.00 (2.01, 4.48)**	**3.36 (1.94, 5.80)**	1.69 (0.52, 5.55)
*p* for trend		<0.001	<0.001	<0.001	0.6
**Uranium**					
<15.7 µg/m^3^	58	1.00 (Reference)	1.00 (Reference)	1.00 (Reference)	1.00 (Reference)
15.7–18.3 µg/m^3^	52	1.07 (0.77, 1.49)	1.07 (0.77, 1.49)	1.01 (0.71, 1.43)	0.84 (0.57, 1.23)
≥18.3 µg/m^3^	64	**1.60 (1.16, 2.20)**	**1.59 (1.15, 2.19)**	1.41 (0.96, 2.08)	0.50 (0.22, 1.16)
Per 5 µg/m^3^	174	**1.53 (1.23, 1.88)**	**1.52 (1.23, 1.88)**	**1.54 (1.15, 2.07)**	0.90 (0.48, 1.68)
*p* for trend		0.002	0.003	0.047	0.084

^a^ Results are in bold where it reached statistical significance (α = 0.05) in a model; **^b^** Adjusted for age, race/ethnicity, BMI, education, and annual family income; **^c^** Adjusted for variables in Model 1, plus smoking status and pack-year; **^d^** Adjusted for variables in Model 2, plus log-transformed NOx; **^e^** Adjusted for variables in Model 2, plus study site; ^f^ Interquartile range for PM_2.5._

**Table A11:** Ratios of geometric means (95% confidence interval) of urinary metals by tertiles of PM_2.5_ concentrations in females ^a^.

PM_2.5_	*N*	Model 1 ^b^	Model 2 ^c^	Model 3 ^d^	Model 4 ^e^
**Antimony**					
<15.7 µg/m^3^	43	1.00 (Reference)	1.00 (Reference)	1.00 (Reference)	1.00 (Reference)
15.7–18.2 µg/m^3^	49	0.83 (0.46, 1.48)	0.80 (0.44, 1.43)	0.87 (0.47, 1.64)	0.92 (0.45, 1.87)
≥18.2 µg/m^3^	38	0.69 (0.36, 1.33)	0.68 (0.35, 1.30)	0.82 (0.37, 1.85)	1.00 (0.29, 3.43)
Per 5 µg/m^3^^f^	130	0.69 (0.43, 1.11)	0.68 (0.42, 1.09)	0.75 (0.41, 1.36)	0.64 (0.23, 1.74)
*p* for trend		0.32	0.28	0.72	0.95
**Cadmium**					
<15.7 µg/m^3^	43	1.00 (Reference)	1.00 (Reference)	1.00 (Reference)	1.00 (Reference)
15.7–18.2 µg/m^3^	49	0.73 (0.50, 1.08)	0.75 (0.52, 1.08)	0.75 (0.50, 1.11)	0.61 (0.40, 0.96)
≥18.2 µg/m^3^	38	0.93 (0.61, 1.44)	1.02 (0.68, 1.55)	1.03 (0.62, 1.70)	0.95 (0.44, 2.03)
Per 5 µg/m^3^	130	0.94 (0.68, 1.29)	1.01 (0.75, 1.37)	1.02 (0.69, 1.50)	0.70 (0.37, 1.31)
*p* for trend		0.99	0.65	0.59	0.68
**Tungsten**					
<15.7 µg/m^3^	43	1.00 (Reference)	1.00 (Reference)	1.00 (Reference)	1.00 (Reference)
15.7–18.2 µg/m^3^	49	1.31 (0.65, 2.64)	1.33 (0.66, 2.70)	1.37 (0.64, 2.92)	1.81 (0.78, 4.21)
≥18.2 µg/m^3^	38	**3.09 (1.42, 6.74)**	**3.17 (1.44, 6.99)**	**3.37 (1.27, 8.90)**	1.18 (0.27, 5.05)
Per 5 µg/m^3^	130	**2.17 (1.23, 3.82)**	**2.21 (1.24, 3.94)**	**2.34 (1.12, 4.86)**	1.24 (0.37, 4.14)
*p* for trend		0.009	0.008	0.034	0.89
**Uranium**					
<15.7 µg/m^3^	43	1.00 (Reference)	1.00 (Reference)	1.00 (Reference)	1.00 (Reference)
15.7–18.2 µg/m^3^	49	1.35 (0.95, 1.90)	1.34 (0.95, 1.90)	1.42 (0.98, 2.07)	1.33 (0.88, 2.02)
≥18.2 µg/m^3^	38	**1.95 (1.33, 2.87)**	**1.92 (1.30, 2.83)**	**2.17 (1.34, 3.50)**	1.06 (0.52, 2.19)
Per 5 µg/m^3^	130	**1.59 (1.20, 2.11)**	**1.57 (1.18, 2.09)**	**1.74 (1.21, 2.49)**	0.97 (0.54, 1.76)
*p* for trend		0.001	0.001	0.001	0.57

^a^ Results are in bold where it reached statistical significance (α = 0.05) in a model; **^b^** Adjusted for age, race/ethnicity, BMI, education, and annual family income; **^c^** Adjusted for variables in Model 1, plus smoking status and pack-year; **^d^** Adjusted for variables in Model 2, plus log-transformed NOx; **^e^** Adjusted for variables in Model 2, plus study site; ^f^ Interquartile range for PM_2.5_.

**Table A12:** Ratios of geometric means (95% confidence interval) of urinary metals by tertiles of NOx concentrations in males ^a^.

NOx	*N*	Model 1 ^b^	Model 2 ^c^	Model 3 ^d^	Model 4 ^e^
**Antimony**					
<37.8 ppb	62	1.00 (Reference)	1.00 (Reference)	1.00 (Reference)	1.00 (Reference)
37.8–69.0 ppb	59	1.21 (0.69, 2.10)	1.21 (0.69, 2.11)	1.18 (0.63, 2.18)	1.64 (0.82, 3.27)
≥69.0 ppb	53	1.63 (0.90, 2.96)	1.68 (0.92, 3.06)	1.60 (0.75, 3.44)	2.65 (1.04, 6.73)
Per 45 ppb ^f^	174	1.27 (0.85, 1.89)	1.29 (0.86, 1.94)	1.20 (0.68, 2.10)	1.52 (0.82, 2.83)
*p* for trend		0.102	0.088	0.21	0.045
**Cadmium**					
<37.8 ppb	62	1.00 (Reference)	1.00 (Reference)	1.00 (Reference)	1.00 (Reference)
37.8–69.0 ppb	59	0.87 (0.59, 1.27)	0.86 (0.60, 1.24)	0.82 (0.55, 1.23)	0.93 (0.59, 1.47)
≥69.0 ppb	53	0.82 (0.54, 1.23)	0.90 (0.61, 1.33)	0.82 (0.50, 1.35)	1.02 (0.55, 1.88)
Per 45 ppb	174	0.86 (0.65, 1.13)	0.93 (0.72, 1.22)	0.86 (0.59, 1.24)	1.01 (0.67, 1.52)
*p* for trend		0.32	0.61	0.46	0.99
**Tungsten**					
<37.8 ppb	62	1.00 (Reference)	1.00 (Reference)	1.00 (Reference)	1.00 (Reference)
37.8–69.0 ppb	59	1.55 (0.82, 2.95)	1.55 (0.81, 2.96)	0.83 (0.42, 1.64)	1.44 (0.68, 3.07)
≥69.0 ppb	53	**3.05 (1.54, 6.06)**	**3.13 (1.56, 6.28)**	1.03 (0.45, 2.39)	2.42 (0.87, 6.70)
Per 45 ppb	174	**2.19 (1.39, 3.45)**	**2.24 (1.41, 3.56)**	0.92 (0.50, 1.71)	1.60 (0.81, 3.15)
*p* for trend		0.001	0.001	0.82	0.081
**Uranium**					
<37.8 ppb	62	1.00 (Reference)	1.00 (Reference)	1.00 (Reference)	1.00 (Reference)
37.8–69.0 ppb	59	1.17 (0.84, 1.64)	1.17 (0.84, 1.64)	0.92 (0.64, 1.32)	0.98 (0.65, 1.47)
≥69.0 ppb	53	**1.51 (1.05, 2.16)**	**1.49 (1.03, 2.14)**	0.96 (0.61, 1.50)	1.03 (0.59, 1.77)
Per 45 ppb	174	**1.42 (1.13, 1.80)**	**1.41 (1.11, 1.80)**	1.05 (0.76, 1.46)	1.14 (0.80, 1.63)
*p* for trend		0.018	0.024	0.99	0.78

^a^ Results are in bold where it reached statistical significance (α = 0.05) in a model; **^b^** Adjusted for age, sex, race/ethnicity, BMI, education, and annual family income; **^c^** Adjusted for variables in Model 1, plus smoking status and pack-year; **^d^** Adjusted for variables in Model 2, plus log-transformed PM_2.5_; **^e^** Adjusted for variables in Model 2, plus study site; ^f^ Interquartile range for NOx.

**Table A13:** Ratios of geometric means (95% confidence interval) of urinary metals by tertiles of NOx concentrations in females ^a^.

NOx	*N*	Model 1 ^b^	Model 2 ^c^	Model 3 ^d^	Model 4 ^e^
**Antimony**					
<37.9 ppb	39	1.00 (Reference)	1.00 (Reference)	1.00 (Reference)	1.00 (Reference)
37.9–68.5 ppb	42	0.79 (0.41, 1.52)	0.82 (0.42, 1.59)	0.90 (0.45, 1.80)	0.76 (0.28, 2.04)
≥69.0 ppb	49	0.65 (0.34, 1.24)	0.66 (0.34, 1.29)	0.84 (0.37, 1.93)	0.51 (0.14, 1.78)
Per 45 ppb ^f^	130	0.67 (0.43, 1.05)	0.69 (0.44, 1.08)	0.77 (0.42, 1.42)	0.36 (0.14, 0.92)
*p* for trend		0.21	0.23	0.68	0.27
**Cadmium**					
<37.9 ppb	39	1.00 (Reference)	1.00 (Reference)	1.00 (Reference)	1.00 (Reference)
37.9–68.5 ppb	42	0.71 (0.46, 1.10)	0.71 (0.47, 1.07)	0.69 (0.45, 1.07)	0.57 (0.30, 1.05)
≥69.0 ppb	49	0.84 (0.55, 1.30)	0.87 (0.57, 1.32)	0.82 (0.49, 1.39)	0.51 (0.23, 1.12)
Per 45 ppb	130	1.01 (0.74, 1.36)	1.02 (0.76, 1.36)	1.02 (0.69, 1.51)	0.82 (0.45, 1.50)
*p* for trend		0.65	0.81	0.72	0.24
**Tungsten**					
<37.9 ppb	39	1.00 (Reference)	1.00 (Reference)	1.00 (Reference)	1.00 (Reference)
37.9-68.5 ppb	42	1.44 (0.65, 3.20)	1.42 (0.63, 3.21)	1.12 (0.48, 2.58)	1.83 (0.56, 5.94)
≥69.0 ppb	49	2.07 (0.93, 4.59)	2.05 (0.91, 4.65)	1.12 (0.41, 3.07)	1.91 (0.43, 8.59)
Per 45 ppb	130	1.47 (0.85, 2.56)	1.45 (0.83, 2.55)	0.80 (0.38, 1.69)	0.77 (0.25, 2.40)
*p* for trend		0.066	0.072	0.64	0.67
**Uranium**					
<37.9 ppb	39	1.00 (Reference)	1.00 (Reference)	1.00 (Reference)	1.00 (Reference)
37.9–68.5 ppb	42	1.18 (0.79, 1.77)	1.18 (0.78, 1.78)	0.97 (0.64, 1.46)	1.24 (0.69, 2.22)
≥69.0 ppb	49	1.22 (0.81, 1.82)	1.20 (0.79, 1.81)	0.73 (0.45, 1.20)	1.06 (0.51, 2.23)
Per 45 ppb	130	1.15 (0.87, 1.52)	1.14 (0.86, 1.51)	0.76 (0.53, 1.08)	0.95 (0.54, 1.66)
*p* for trend		0.4	0.47	0.14	0.89

^a^ Results are in bold where it reached statistical significance (α = 0.05) in a model; **^b^** Adjusted for age, sex, race/ethnicity, BMI, education, and annual family income; **^c^** Adjusted for variables in Model 1, plus smoking status and pack-year; **^d^** Adjusted for variables in Model 2, plus log-transformed PM_2.5_; **^e^** Adjusted for variables in Model 2, plus study site; ^f^ Interquartile range for NOx.

**Table A14:** Ratios of geometric means (95% confidence interval) of urinary metals by tertiles of NOx concentrations ^a^.

NOx	*N*	Model 1 ^b^	Model 2 ^c^	Model 3 ^d^	Model 4 ^e^
**Antimony**					
<37.9 ppb	101	1.00 (Reference)	1.00 (Reference)	1.00 (Reference)	1.00 (Reference)
37.9–69.0 ppb	101	0.95 (0.63, 1.42)	0.97 (0.64, 1.45)	1.00 (0.64, 1.56)	1.19 (0.69, 2.04)
≥69.0 ppb	102	1.04 (0.68, 1.59)	1.05 (0.68, 1.62)	1.11 (0.64, 1.93)	1.36 (0.66, 2.79)
Per 45 ppb ^f^	304	0.94 (0.71, 1.26)	0.94 (0.70, 1.27)	0.92 (0.62, 1.38)	0.95 (0.58, 1.56)
*p* for trend		0.82	0.79	0.66	0.42
**Cadmium**					
<37.9 ppb	101	1.00 (Reference)	1.00 (Reference)	1.00 (Reference)	1.00 (Reference)
37.9–69.0 ppb	101	0.82 (0.62, 1.08)	0.82 (0.63, 1.06)	0.78 (0.58, 1.04)	0.83 (0.59, 1.18)
≥69.0 ppb	102	0.86 (0.64, 1.14)	0.92 (0.70, 1.22)	0.84 (0.59, 1.20)	0.93 (0.59, 1.48)
Per 45 ppb	304	0.93 (0.76, 1.13)	0.98 (0.81, 1.19)	0.93 (0.72, 1.21)	1.06 (0.77, 1.46)
*p* for trend		0.38	0.74	0.52	0.99
**Tungsten**					
<37.9 ppb	101	1.00 (Reference)	1.00 (Reference)	1.00 (Reference)	1.00 (Reference)
37.9–69.0 ppb	101	1.48 (0.92, 2.38)	1.49 (0.92, 2.40)	0.94 (0.57, 1.57)	1.51 (0.82, 2.78)
≥69.0 ppb	102	**2.61 (1.58, 4.32)**	**2.69 (1.62, 4.48)**	1.15 (0.62, 2.15)	**2.40 (1.07, 5.41)**
Per 45 ppb	304	**1.84 (1.31, 2.59)**	**1.87 (1.32, 2.65)**	0.90 (0.57, 1.43)	1.37 (0.78, 2.40)
*p* for trend		<0.001	<0.001	0.56	0.036
**Uranium**					
<37.9 ppb	101	1.00 (Reference)	1.00 (Reference)	1.00 (Reference)	1.00 (Reference)
37.9–69.0 ppb	101	1.15 (0.90, 1.47)	1.15 (0.90, 1.47)	0.90 (0.70, 1.17)	1.03 (0.76, 1.41)
≥69.0 ppb	102	**1.33 (1.03, 1.72)**	**1.31 (1.01, 1.70)**	0.84 (0.61, 1.15)	1.05 (0.69, 1.58)
Per 45 ppb	304	**1.28 (1.07, 1.52)**	**1.26 (1.06, 1.51)**	0.90 (0.71, 1.14)	1.10 (0.82, 1.46)
*p* for trend		0.031	0.044	0.30	0.85

^a^ Results are in bold where it reached statistical significance (α = 0.05) in a model; **^b^** Adjusted for age, sex, race/ethnicity, BMI, education, and annual family income; **^c^** Adjusted for variables in Model 1, plus smoking status and pack-year; **^d^** Adjusted for variables in Model 2, plus log-transformed PM_2.5_; **^e^** Adjusted for variables in Model 2, plus study site; ^f^ Interquartile range for NOx.
